# Characterization of a fatal feline panleukopenia virus derived from giant panda with broad cell tropism and zoonotic potential

**DOI:** 10.3389/fimmu.2023.1237630

**Published:** 2023-08-17

**Authors:** Shan Zhao, Huanyuan Hu, Jingchao Lan, Zhisong Yang, Qianling Peng, Liheng Yan, Li Luo, Lin Wu, Yifei Lang, Qigui Yan

**Affiliations:** ^1^ College of Veterinary Medicine, Sichuan Agricultural University, Chengdu, China; ^2^ Key Laboratory of Animal Disease and Human Health of Sichuan Province, Sichuan Agricultural University, Chengdu, China; ^3^ Chengdu Research Base of Giant Panda Breeding, Chengdu, China; ^4^ Sichuan Academy of Giant Panda, Chengdu, China

**Keywords:** feline panleukopenia virus, giant panda, cross-species transmission, TfR, fatal

## Abstract

Represented by feline panleukopenia virus (FPV) and canine parvovirus (CPV), the species *carnivore protoparvovirus 1* has a worldwide distribution through continuous ci13rculation in companion animals such as cats and dogs. Subsequently, both FPV and CPV had engaged in host-to-host transfer to other wild animal hosts of the order *Carnivora*. In the present study, we emphasized the significance of cross-species transmission of parvoviruses with the isolation and characterization of an FPV from giant panda displaying severe and fatal symptoms. The isolated virus, designated pFPV-sc, displayed similar morphology as FPV, while phylogenetic analysis indicated that the nucleotide sequence of pFPV-sc clades with Chinese FPV isolates. Despite pFPV-sc is seemingly an outcome of a spillover infection event from domestic cats to giant pandas, our study also provided serological evidence that FPV or other parvoviruses closely related to FPV could be already prevalent in giant pandas in 2011. Initiation of host transfer of pFPV-sc is likely with association to giant panda transferrin receptor (TfR), as TfR of giant panda shares high homology with feline TfR. Strikingly, our data also indicate that pFPV-sc can infect cell lines of other mammal species, including humans. To sum up, observations from this study shall promote future research of cross-host transmission and antiviral intervention of *Carnivore protoparvovirus 1*, and necessitate surveillance studies in thus far unacknowledged potential reservoirs.

## Introduction

Parvoviruses (family *Parvoviridae*) are a group of small, non-enveloped, single-stranded DNA viruses. They have a linear DNA genome that are about 4.5–5.5 kb in length, with hairpin structures composed of inverted terminal repeat (ITR) folds at both ends of the viral genome (1). The rest of the viral genome contains two major open reading frames which encode two non-structural proteins (NS1, NS2) and two structural proteins (VP1, VP2) in the same mRNA through variable splicing (2, 3). Parvoviruses are currently endemic worldwide and can naturally infect a wide range of hosts (4–9). Due to its characteristics of rapid evolution and transmission, the host spectrum of parvovirus is still expanding, posing a threat on a variety of endangered wild animal species.

*Carnivore protoparvovirus 1* is a distinctive species under the *Protoparvovirus* genus of family *Parvoviridae (* 1). Members of this species include feline panleukopenia virus (FPV), canine parvovirus (CPV), mink enteritis virus (MEV) and raccoon dog parvovirus (RaPV) (4, 10–12). Both FPV and CPV have a worldwide distribution in companion animals, causing symptoms such as vomiting, severe diarrhea and leukopenia (13, 14). Importantly, FPV and CPV do not limit themselves in cats and dogs. In recent years, infection with both virus were recurrently reported in wild animals such as giant panda, raccoon, African lion, leopard and white tiger (15–20).

Host range is a key distinctive of viruses, which determines the host specificity and also reflects the diversity of natural host tropism. Virus host range expansion often provides a certain basis for the emergence of new diseases. In the case of carnivore protoparvovirus 1, studies showed that transferrin receptor (TfR) is the main host receptor that mediates virus binding and cellular entry (21–23). TfR is a dimeric membrane-associated protein located on the cell surface, which functions through binding and importing iron particles of transferrin-mediated cell intake of iron ions (21). As the parvoviral receptor, TfR interacts with the virus capsid through a compartment that appears to center around residue 300 of the viral VP2 protein, but other rather distanced VP2 residues are also involved, suggesting a broad interaction with the viral capsid (24–26). Consequently, such machnism shall allow viruses to bind TfRs of other carnivores, hence initiate cross-species virus transmission (27).

In the present study, we confirmed the multi-host nature of carnivore protoparvovirus 1 with isolation and characterization of an FPV in giant pandas from Chengdu Research Base of Giant Panda Breeding. Genetic and biological features of this virus were studied, and the findings revealed the zoonotic threats of this virus and *carnivore protoparvovirus 1* in general.

## Materials and methods

### Fecal and serum samples from giant panda

Giant panda fecal samples were collected from captive giant pandas at Chengdu Research Base of Giant Panda Breeding in 2020. All of the sampled giant pandas (n=15) displayed severe symptoms such as diarrhea, vomiting and anorexia, while two juvenile pandas were dead before effective treatment. Serum samples (n=14) were retrieved from the serum bank of our lab, which were sera from 14 different healthy captive giant pandas between 2010 and 2018. All samples were stored at -80°C before usage.

### Cells

Feline kidney cells (F81), Crandell-Rees Feline Kidney (CRFK), African green monkey kidney cells (Vero-CCL81), African Green monkey foetal kidney (MA-104), porcine kidney epithelial cells (LLC-PK1), porcine endothelial cells (PIEC), human hepatoma cells (Huh7), human rectal Adenocarcinoma cells (HRT-18), Hela and human embryonic kidney 293 cells stably expressing the SV40 large T antigen (HEK-293T) were maintained in Dulbecco modified Eagle medium (DMEM, Gibco) supplemented with 10% fetal bovine serum (FCS), penicillin (100 IU/mL) and streptomycin (100 μg/mL).

### Detection of parvovirus infection and virus isolation

Total DNA of giant panda fecal samples were extracted using the RoomTempTM sample lysis kit (Vazyme, P073) or Stool DNA Isolation Kit (Foregene, DE-05713) per the instructions of the manufacturer. Two primer pairs targeting the conserved ORF1 region of parvovirus genomes, namely P1F 5’-AACAAGCAACTGGTAAATGGCT-3’, P1R 5’-CACAGCTTGTGCTATGGCTTGA-3’, P2F 5’-TCACGCTATAGCATGTGTTTTA-3’ and P2R 5’-ACTAACACACCCTTACCTCTCC-3’, were used to detect traces of parvovirus infection. Polymerase chain reaction (PCR) was carried out by GoTaq^®^ DNA Polymerase (Promega, M3001). The PCR procedure was as follows: 95°C for 3 min, followed by 34 cycles of 95°C for 15 s, 51°C (P1) or 47°C (P2) for 15s, and 72°C for 15s, with a final extension at 72°C for 5 min. For virus isolation, PCR-positive samples were homogenized in phosphate buffered saline (PBS), filtrated through 0.22μm filters and inoculated onto monolayers of F81 cells. Four hours post inoculation, the inoculum was replaced with virus growth medium (DMEM supplemented with 2% FCS and 10mM HEPES) and incubated at 37°C, 5% CO_2_. Supernatants from inoculated cells were then blind passaged in F81 cells until cytopathic effect (CPE) started to appear. Viruses were then titrated and purified by end-point dilution on F81 cells with CPE as read-out. Titer of the propagated clonal virus, designated as pFPV-SC, was calculated by the Spearman–Kaerber formula. Morphology of the virus particles was observed via transmission electron microscopy post ultracentrifugation and negative staining with 0.5% phosphotungstic acid.

### Genetic and growth characterization of the isolated virus

To obtain complete genetic information of the isolated virus, multiple overlapped PCRs were performed using a set of primers designed in the present study after analysis of different parvoviral genomes (detailed in **Table 1**). PCR products were cloned with CloneJET PCR Cloning Kit (Thermo Fisher, K1231) followed by conventional bidirectional sanger sequencing. Deduced viral sequences were assembled via DNASTAR Lasergene software package to acquire the full viral genome. To determine viral growth kinetics, F81 cells were infected with the isolated parvovirus at a multiplicity of infection (MOI) of 0.01. Cell culture medium supernatants were harvested every 12 hours from 0 to 72 hours post-infection (hpi). Viral titers (TCID_50_/mL) were determined by end-point dilution with F81 cells as described above. Experiments were performed in independent triplicates, and the calculated values are expressed as the mean ± standard deviation.

**Table 1:** Primers used to amplify the genome sequences of the isolated parvovirus.

Primer pair	Sequence (5’-3’)	Position	Target (nt)
1	F: ATCATTCTTTAGAACCAACT R: GGTGTTAAGTTTACCGAACA	1-20 778-759	759
2	F: GTACGTATGACGTGATGACGC R: ATTCACTATCTTCTGCAATTT	33-53 822-802	770
3	F:CACACGTCATACGTACGCTCCT R: GTACGTATGACGTGATGACGC	80-101 856-835	756
4	F:TAAAGAATGATAGGCGGTTTGTGT R: TTTGTCTGTCTTGATACTTCATAA	116-139 1014-991	876
5	F:AACAAGCAACTGGTAAATGGCT R: CACAGCTTGTGCTATGGCTTGA	691-712 1523-1502	812
6	F:TCACGCTATAGCATGTGTTTTA R: ACTAACACACCCTTACCTCTCC	1409-1430 2332-2311	793
7	F:GAAGATTTTCGAGACGACTTGGAT R:TACTATCTAATGCAACCATCAATG	2253-2276 3301-3324	1049
8	F: GAGATTGGCAACTAATTGTTAA R:TTATTTAATGCAGTTAAAGGAC	3157-3178 4162-4141	985
9	F:CAGGAAGATATCCAGAAGGAGA R:CAACCACCCACACCATAACAAC	4003-4024 4800-4821	780
10	F:TATCAACTAGCACCTAGAAAATTA R: AAGTATCAATCTGTCTTTAAGGGG	4572-4595 5100-5123	528
11	F:TATTAATGTATGTTGTTATGGTGT R:AGATACACAACATCAGTAGACTGA	4789-4812 5087-5064	276

Nucleotide positions were marked based on the reference strain HH-1/86.

### Animal studies with the isolated parvovirus

To facilitate research described in this study, hyperimmune serum was collected from an inoculated rabbit. Briefly, a 4-week-old gnotobiotic rabbit was subcutaneously injected with two consecutive doses of heat-inactivated virus (14 days interval; 10^4^ TCID_50_/dose), and hyperimmune serum was collected 14 days post the second injection. The animal use protocols commissioned in this study were reviewed and approved by the Animal Ethics Committee of Sichuan Agricultural University.

### Phylogenetic analysis

Sequence containing the complete viral genome, assembled by the DNASTAR Lasergene software package, were further processed by the online tool Clustal Omega (https://www.ebi.ac.uk/Tools/msa/clustalo/). Multiple alignments of the full-length genomes and VP2 genes with representative parvovirus sequences and phylogenetic analyses were then conducted using the neighbor-joining method in MEGA X, respectively.

### Virus neutralization assay

Neutralization assays were performed with pFPV-sc to examine the seropositivity of giant panda serum samples. Different samples were two-fold serially diluted in virus growth medium, and mixed 1:1 with pFPV-sc (2000 TCID_50_/mL). The mixtures were incubated at 37°C for one hour, and 100 μL of each mixture was used for inoculation onto F81 cells. At 3–5 days post infection, CPE was observed by microscopy. Virus neutralization titers (VNT) were expressed as the highest serum dilution resulting in 90% reduction of CPE. Experiments were performed in independent triplicate.

### Hemagglutination and hemagglutination inhibition assay

Hemagglutination (HA) assay was performed with giant panda, human, rat, pig and rabbit erythrocytes (0.5% suspension in PBS). Two-fold serial dilutions of viruses, starting at 10^4^ TCID_50_ per well, were mixed 1:1 with the erythrocytes in a V-shape plate (Corning™, 3894). Hemagglutination was assessed after 2-hour incubation on ice, and hemagglutinating units (HAU) were inspected for erythrocytes of each species. For desiaylation, erythrocytes were treated with *Arthrobacter ureafaciens* neuraminidase (Roche, 10mU/mL) for 3 hours at 37°C, and washed 5 times with cold PBS prior to the HA assay.

Hemagglutination inhibition (HI) assays were carried out to study the HA inhibition ability of different serum samples. Different samples were two-fold serially diluted, and mixed 1:1 with PBS containing 8 HAU of pFPV-sc. The mixtures were incubated at room temperature for one hour, and then mixed 1:1 with human erythrocytes (0.5% in PBS). Hemagglutination inhibition was documented after 2-hour incubation on ice. The HI titer of one serum sample is conveyed as the reciprocal of the highest serum dilution that still shows hemagglutination inhibition.

### Immunofluorescence assay

An immunofluorescence assay (IFA) was carried out to examine the susceptibility of different cell lines to pFPV-sc. Briefly, cells on 24-well plates were infected with the virus at a MOI of 0.1, then incubated at 37°C, 5% CO_2_ prior to washing with PBS and fixation with 4% Paraformaldehyde. Cells were then blocked with PBS containing 2% bovine serum albumin (Sigma), and incubated with 200 μl rabbit hyperimmune serum at a 1:150 dilution. One hour later, cells were washed three times with PBS, followed by another incubation step with 200 μl FITC-conjugated goat anti-rabbit IgG antibody (Thermo Fisher Scientific) at 1:400 dilution mixed with 4′, 6-diamidino-2-phenylindole (DAPI) at 1:1000 dilution. Lastly, cells were washed, mounted on glass slides and visualized under a fluorescence microscope.

### Modelling of the giant panda transferrin receptor

Structure homology-modeling of transferrin receptors (TfRs) of giant panda (NCBI reference sequence: XM_011221715.3) and cat (GenBank accession number: AF276984.1) was performed by the SWISS-MODEL server (https://www.swissmodel.expasy.org/) using the deciphered structure of human TfR (PDB accession number: 7ZQS) as a search model.

## Results

### Isolation and characterization of a feline panleukopenia virus from captive giant panda

**Figure 1:**
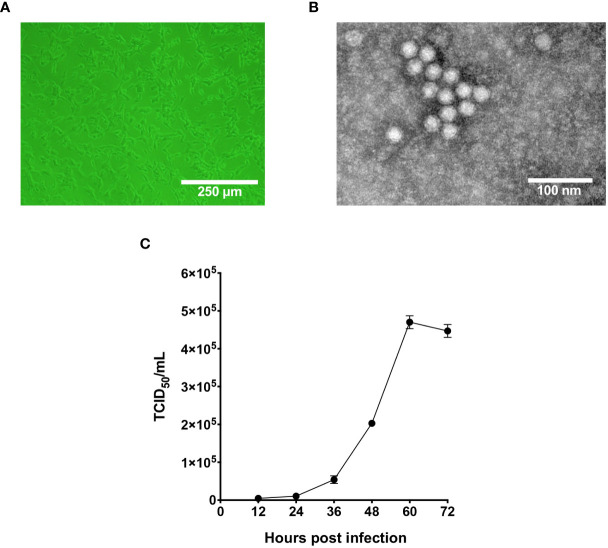
Characterization of the infectivity and morphology of pFPV-sc. **(A)** Cytopathic effect induced by pFPV-sc (clonal virus population) in F81 cells. **(B)** Morphology of pFPV-sc particles exhibited with negative-stained transmission electron microscopy. **(C)** Growth curve of pFPV-sc as measured by end-point dilution. Each datapoint shows averages of three independent replicates, and standard deviations are indicated as error bars.

In 2020, captive giant pandas from Chengdu Research Base of Giant Panda Breeding were reported to display fatal symptoms such as diarrhea and vomiting, and two giant panda cubs did not survive the infection. Fecal samples from those pandas were tested pan-parvovirus positive by conventional PCR. To further validate the infection status, fecal samples were inoculated on F81 cells for probable virus isolation. As shown in **Figure 1A**, after five blind passages in F81 cells, typical parvovirus-induced CPE was formed, indicated by cell rounding, pyknosis, disturbance of cell monolayers and eventually full necrosis. At passage 10, the titer of the nonclonal virus population had reached 1x10^6^ TCID_50_/mL measured by end-point dilution assay. TEM analysis of negative stained, purified cell supernatant showed spherical particles with an average diameter around 20 nm, which falls to the typical parvovirus morphology (**Figure 1B**). To study the cellular infectivity of the isolated parvovirus in detail, the mixed viral population was further purified and propagated, and growth kinetics was analyzed of the clonal virus, designated pFPV-sc. The results indicated that the viral growth took a steady start, and peaked at 60 hours post infection (**Figure 1C**).

### pFPV-sc is genetically closely related to FPV than CPV

**Figure 2:**
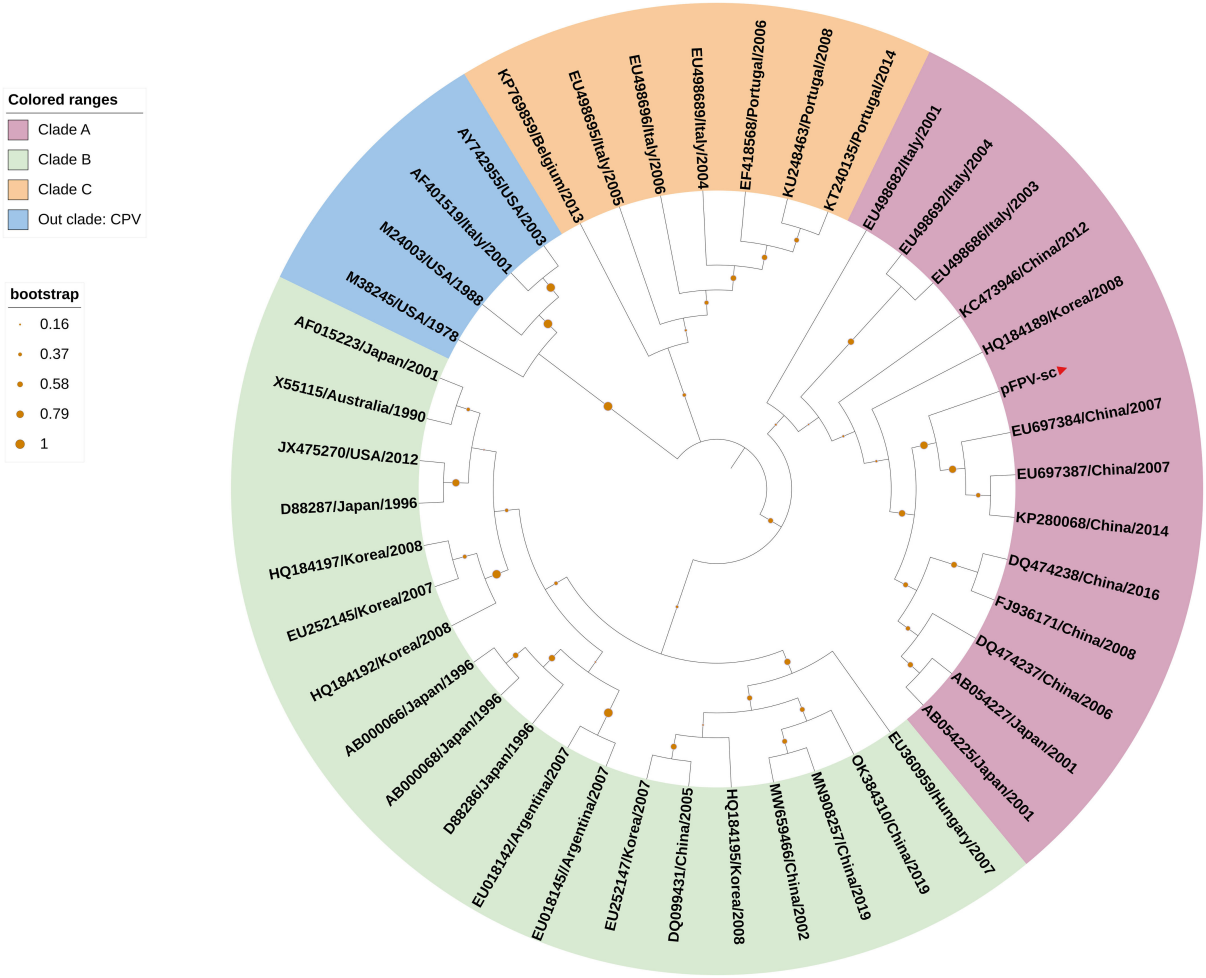
VP2-based phylogenetic analysis indicates pFPV-sc clades with recent FPV strains. The tree was generated based on full-length VP2 sequence of different FPVs using the neighbor-joining method with the Jukes-Cantor algorithm of distance correction, with bootstrapping up to 1000 replicates. GenBank accession numbers are specified for each reference strain. The three FPV clades, namely clade A, B and C, were highlighted with different colors, with the branch of pFPV-sc highlighted in red. CPV was used as out group.

To acquire the complete genetic information of this isolated virus, the viral genome was amplified in several fragments via PCR followed by Sanger sequencing (**Table 1**). Noticeably, sequences of the long inverted terminal repeats (ITR) at both the 5’ and 3’ end of the viral genome were obtained with two overlapping PCRs, of which primers were designed based on the conserved regions of different parvovirus genomes. After validation of sequencing results, it is confirmed that the full-length genome of the isolate pFPV-sc was amplified and sequenced. The genome is of 5,119 nucleotides in length, which encompasses the two ITRs and encoding regions for the nonstructural protein (NS) and two capsid proteins (VP1 and VP2). The full genome information is deposited to Genbank (OR264206). The 5’ and 3’ ITR form two imperfect palindromes typical for parvoviruses, and their sequences are identical with reference FPV strain HH-1/86 (28). In the meantime, the NS gene of this isolate exhibited similar identities to FPV, CPV and mink enteritic virus (99.1%–100%). To gain further insight into the antigenicity of this isolate, multiple sequences alignment analysis was performed on the nucleotide and translated amino acid sequences of the VP2 gene. The results indicated that the VP2 gene shared higher identities with FPV (98.8%-99.4%) than CPV (96.4%-98.5%). Noticeably, the VP2 sequence of pFPV-sc presents a unique amino acid substitution His^234^Tyr, which is not found in cat FPVs but in a FPV strain isolated from leopard. The phylogenetic tree based on the VP2 nucleotide sequences showed that pFPV-sc is located in clade A, which is composed by representative FPV strains recently isolated from China and other countries (**Figure 2**).

### pFPV-sc exhibits hemagglutination activity against selected erythrocytes

**Figure 3:**
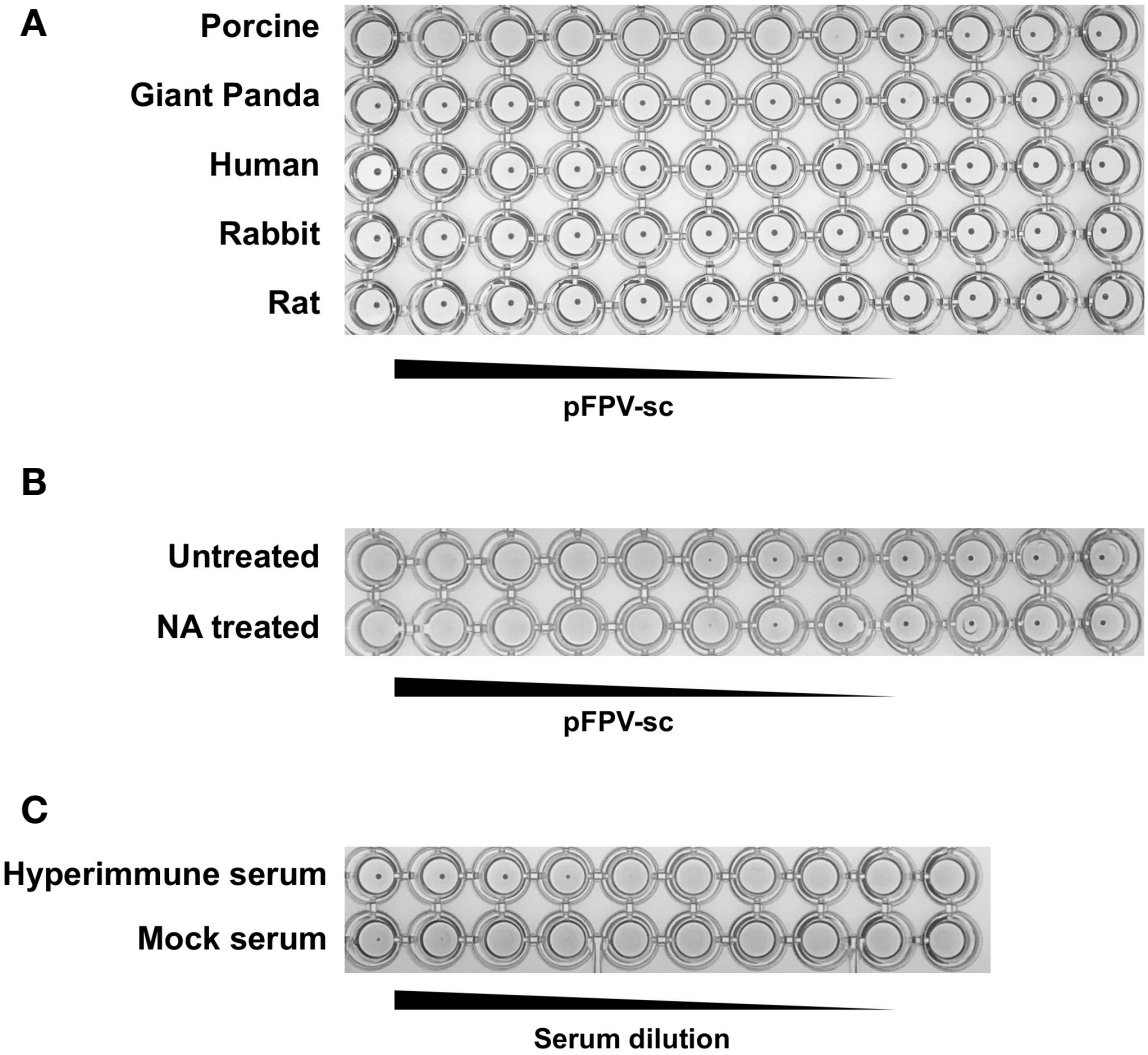
pFPV-sc has specific hemagglutination ability towards porcine erythrocytes. **(A)** Hemagglutination assay (HAA) of pFPV-sc performed with giant panda, human, rat, pig and rabbit erythrocytes. Twofold serial dilutions of pFPV-sc, starting at 10^4^ TCID_50_ per well, were mixed 1:1 with 0.5% erythrocytes diluted in PBS. Hemagglutination was assessed after 2 hour incubation on ice. HAAs were repeated at least three times and representative experiments are shown. **(B)** Hemagglutination of pFPV-sc is not sensitive to neuraminidase (NA) treatment. Untreated and NA (from *Arthrobacter ureafaciens*) treated porcine erythrocytes were compared via HAA as in **(A)**, and NA treatment does not affect the outcome. **(C)** Inhibition of pFPV-sc hemagglutination with hyperimmune serum. Hemagglutination inhibition assay was twofold serial dilutions of unimmunized serum and immunized hyperimmune serum mixed with 8 hemagglutination units of pFPV-sc.

Next, the ability of the isolated pFPV-sc to cause hemagglutination was examined. Erythrocytes from different species, including giant panda, human, rabbit, pig and rat, were included in the analysis. Strong hemagglutination against pig erythrocytes was observed, but not with erythrocytes of other species (**Figure 3A**). At 1x10^5^ TCID_50_/mL, the virus exhibited more than 128 hemagglutination units. Noticeably, treatment of the erythrocytes with *A.ureafaciens* neuraminidase did not hamper hemagglutination (**Figure 3B**), suggesting that the hemagglutination is likely not mediated by virus binding to 5-N-aceylated-sialic acids. To allow confirmation of the specificity of virus-erythrocytes interaction, we embarked on an hemagglutination inhibition (HI) assay with hyperimmune serum collected from an immunized rabbit. As shown in **Figure 3C**, the hyperimmune serum displays prominent HI titer against the virus, while mock immunized serum did not show inhibition. Therefore, the results confirmed that the interaction between the virus and erythrocytes is specific.

### Serologic evidence of early prevalence of parvoviruses in giant panda

**Figure 4:**
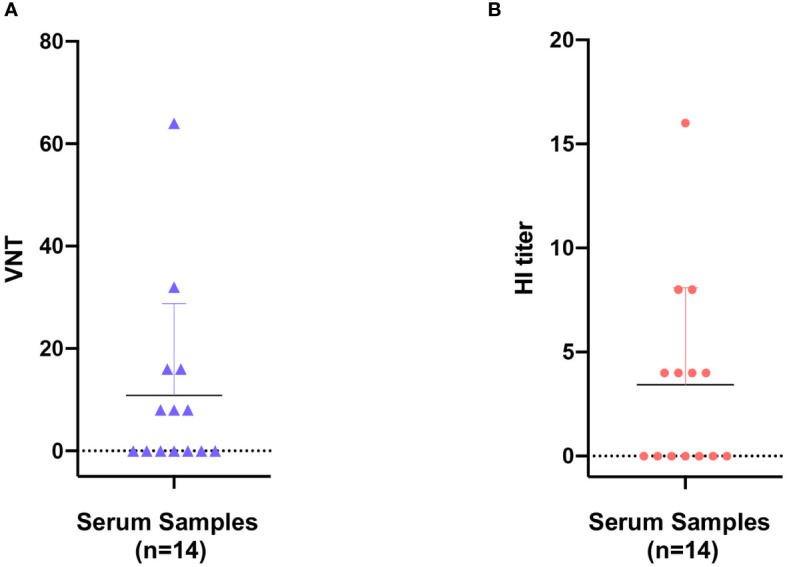
Seroprevalence of parvovirus antibodies in giant panda samples from 2010 to 2018. Reactivity of giant panda serum samples (n=14) against pFPV-sc were measured by virus neutralization assay **(A)** and hemagglutination inhibition (HI) assay **(B)**. Reactivity profiles of all serum samples are displayed as distribution dot plots, with each data point represents the virus neutralization titer (VNT) or HI titer of a particular sample.

Here in the present study, the pathogenetic virus pFPV-sc is newly isolated, however the possibility remains that FPV is already present in the giant panda population through continuous circulation. To this end, a series of giant panda serum samples collected at various time points were tested via different serological methods, with the earliest sample collected in 2011. The virus neutralization titer (VNT) and HI titer of positive samples varied from 8 to 64 and 4 to 16, respectively (**Figure 4**). Within the total 14 serum samples, one particular sample collected in 2011 displayed the highest VNT and HI titer. Despite no clear pattern was observed, our data provided evidence for early prevalence of parvoviruses in the giant panda population in early 2010’s.

### pFPV-sc can infect Cells other than feline origin

**Figure 5:**
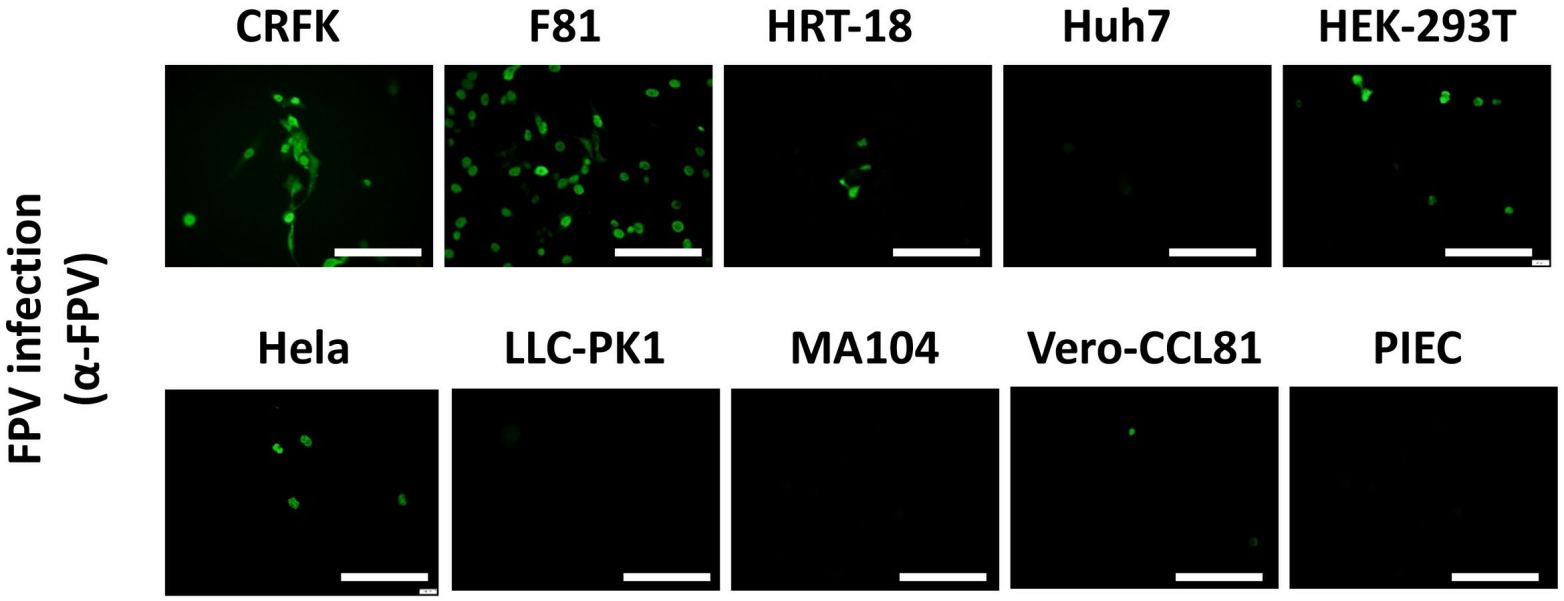
pFPV-sc can infect cells other than feline origin. Immunofluorescent staining was performed with hyperimmune serum upon pFPV-sc infected human, feline, porcine and African green monkey cell lines. Green fluorescence indicates infection; scale bars, 250 μm. Note that three out of four human cell lines tested (HRT-18, HEK-293T and Hela) are susceptible to pFPV-sc. This panel shows representative micrographs from at least three repeats.

The family parvovirus has a relatively wide host spectrum and colonizes a lot of species. To inspect on the cross-species transmission potential of pFPV-sc, we investigated the susceptibility of cell lines from species other than cat (as there is no giant panda cell lines available). Several human, feline, porcine and monkey cell lines were inoculated with viruses at a MOI = 0.1 for 1 h, and infection levels were assessed by immunostaining with the hyperimmune serum (**Figure 5**). Strong fluorescence was noticed in infected CRFK cells, indicated that pFPV-sc can replicate to similar levels in CRFK as in F81 cells (**Figure 5**). Strikingly, Vero-CCL81 and three out of four human cell lines tested (HRT-18, HEK-293T and Hela) also showed susceptibility of PFPV-SC to a particular level (**Figure 5**).

### Giant panda transferrin receptor as a putative receptor for pFPV-sc

**Figure 6:**
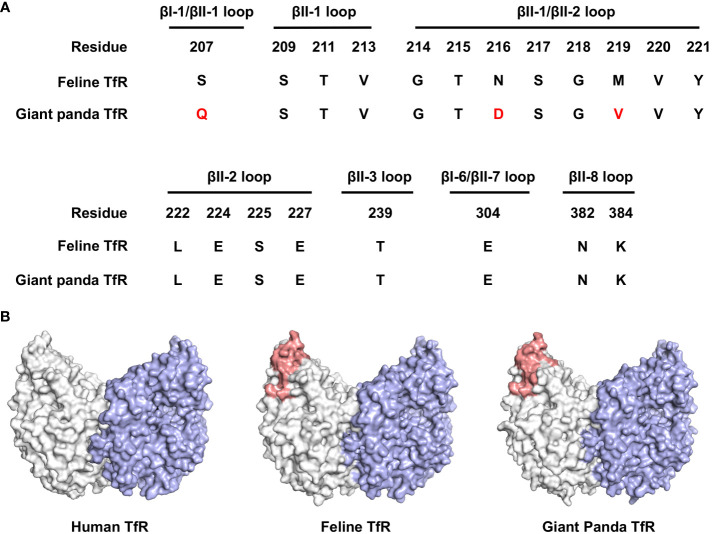
Bioinformatical and structural analysis indicates giant panda transferrin receptor (TfR) as a putative receptor for pFPV-sc. **(A)** Giant panda TfR is akin to the feline TfR at the virus-binding motif. Multiple sequence alignment indicated that the majorities of the crucial amino acid residues that forms the virus binding motif (βI-1, βII-1, βII-2, βII-3, βI-6, βII-7 and βII-8 loops) are conserved between feline and giant panda TfRs. Differences between giant panda TfR and feline TfR are marked red. **(B)** TfR topology are relatively conserved between different host species, with the virus binding motif well exposed. Side view of the dimeric human TfR (PDB accession number: 7ZQS), feline TfR and giant panda TfR (modelled using homology modelling) are shown in surface representation and colored by one monomer gray, and another monomer blue. Virus binding motifs of feline and giant panda TfRs, specified in **(A)**, were depicted in red in the gray-colored monomer.

Carnivore protoparvovirus 1 utilizes transferrin receptor (TfR) as their main receptor for cellular entry, and attachment to TfR of a different species is the starting point of parvovirus cross-species transmission (27). To this end, we evaluated the molecular properties of the predicted giant panda TfR ortholog in comparison with feline TfR. Alignment of the primary sequences shows that the two TfRs share 87.7% identity, while the amino acid residues involved in virus binding are mainly conserved (**Figure 6A**). Next, using homology modeling, we showed that the topology of giant panda TfR is similar to feline TfR and human TfR, where the residues that likely induce virus binding are conserved and exposed on the edge of TfR molecule (**Figure 6B**). Such topology shall allow attachment of the virus, henceforth mediate viral entry.

## Discussion

*Carnivore protoparvovirus 1* can infect a wide range of domestic and wild carnivores, making it a worldwide endemic virus with a broad host range (9, 18). Researchers had identified feline panleukopenia virus (FPV) and canine parvovirus (CPV) infection in different species such as raccoon, African lion, leopard and white tiger, but infection in giant pandas had only received limited attention (15–20). We here isolated an FPV (pFPV-sc) from giant panda displaying severe and even lethal symptoms, and provided the full genome information for a giant panda derived parvovirus for the first time. The damage of this virus caused to giant panda, together with its unique features and the ability of this isolate to infect cells of different mammalian species warrant further investigation into the virus’ characteristics and transmissibility across host spectrums.

Transmission of Carnivore protoparvovirus 1 to other (wild) animal species largely occurs through indirect contact with fomites like feces or saliva from domestic animals. Over the past decades, acute or persistent infections of FPV were reported in wild animals such as lion, leopard and white tiger, leading to symptoms from mild to lethal (16, 18, 19). Domestic or stray cats were proven to be the source of infection in most cases. Here we demonstrate that the isolated giant panda derived pFPV-sc virus shares high identities with Chinese FPV strains, as shown by phylogenetic analysis with the complete genome and the VP2 encoding region. Observations as such indicate that cats were the probable cause of infection, where the workers involved in giant panda breeding might have direct or indirect contact with FPV-infected cats. The isolated pFPV-sc is most likely the result of a spillover infection, as no sign of adaptive mutations were detected. However, our observations do not completely rule out the possibility of FPV persistent infection in giant panda. Serological evidence indicates that giant panda serum samples collected from 2011 are FPV seropositive, suggesting that continuous circulation or repeated infection might occur. Therefore, recurrent epidemiological surveillance is important to monitor possible sustained transmission of Carnivore protoparvovirus 1 infection in giant panda and a wider range of other wild animal species, which could serve as an early cautioning system for imminent threats.

Receptor interaction is the very first and essential step in virus infection of host cells. Therefore, receptor specificity has a direct impact on virus host tropism, and effective virus cross-species transmission depends on the ability of functional utilization of a receptor of an alternative host. In the case of *Carnivore protoparvovirus 1*, transferrin receptor (TfR) serves as its receptor and affinity of TfR association determines both the antigenicity and host range (22, 24, 27). In our analysis, we showed that giant panda TfR shares high similarity with feline TfR, especially the virus interacting motif (26) is highly conserved. This resembles the classic pathway of virus host switching and virus speciation, where binding to giant panda TfR by FPV could initiate infection. Direct studies on the interaction mechanism *in vitro* between FPV and giant panda TfR may reveal the molecular basis for this particular receptor usage.

Besides presumed utilization of giant panda TfR, the isolate pFPV-sc is also shown to be able to agglutinate erythrocytes of certain species. For most viruses, ability of inducing hemagglutination means preferential binding to sialic acids, a group of nine carbon sugars that commonly forms the terminal residues of glycoconjugates (29). Due to its unique topology, binding to sialic acids will allow enhanced adhesion of the virus to the cell surface, hence in closer proximity to the main receptor (29–31). Noticeably, the hemagglutination activity of the isolate pFPV-sc is resistant to neuraminidase treatment, indicating that it might bind to N-glycolylneuraminic acid (Neu5Gc), which correlates with previous findings (32). To this point, the exact machnism and the evolutional advantage in virus fitness upon Carnivore protoparvovirus 1-sialic acid association is still remains to be appreciated.

Despite a successful host jump from domestic cats to giant panda, one of our most striking observations is that pFPV-sc can also infect different cell lines from other mammal species, including humans. Whether susceptibility of those cell lines is due to virus binding to their TfR orthologues or a yet unidentified (co-) receptor, is still unclear. Meanwhile, this finding is currently limited to the level of tissue cultured cells, while infection models build upon organoids or genetically engineered lab animals might collectively reveal the proneness of pFPV-sc to other potential hosts.

Taken together, our observations emphasized the damage of pFPV-sc to the giant panda population, and also underscored the multi-host potential of *Carnivore protoparvovirus 1* in general. Future studies shall focus on the infection machnism and antiviral intervention of *Carnivore protoparvovirus 1*, as its global distribution in domestic and wild animals with cross-species potential is alarming from an epidemiological perspective.

## Data availability statement

The original contributions presented in the study are included in the article/supplementary material, further inquiries can be directed to the corresponding authors.

## Ethics statement

The animal study was approved by Animal Ethics Committee of Sichuan Agricultural University. The study was conducted in accordance with the local legislation and institutional requirements.

## Author contributions

SZ, JL, HH and ZY carried out the experiments. YL and QY supervised the study. YL and SZ contributed to the conception of this article. SZ, JL, HH, ZY, LY, QP, LL, LW, YL and QY analysed data. SZ and YL wrote the manuscript with input from all authors. All authors contributed to the article and approved the submitted version.

## References

[1] Cotmore SF Agbandje-McKenna M Canuti M Chiorini JA Eis-Hubinger AM Hughes J. ICTV virus taxonomy profile: parvoviridae. J Gen Virol (2019) 100:367. doi: 10.1099/jgv.0.001212 30672729 PMC6537627

[2] Reed AP Jones EV Miller TJ. Nucleotide sequence and genome organization of canine parvovirus. J Virol (1988) 62:266. doi: 10.1128/jvi.62.1.266-276.1988 2824850 PMC250527

[3] Chung HC Kim SJ Nguyen VG Shin S Kim JY Lim SK. New genotype classification and molecular characterization of canine and feline parvoviruses. J Vet Sci (2020) 21. doi: 10.4142/jvs.2020.21.e43 PMC7263909 32476317

[4] Parrish CR. Emergence, natural history, and variation of canine, mink, and feline parvoviruses. Adv Virus Res (1990) 38:403. doi: 10.1016/S0065-3527(08)60867-2 2171302 PMC7131698

[5] Parrish CR Have P Foreyt WJ Evermann JF Senda M Carmichael LE. The global spread and replacement of canine parvovirus strains. J Gen Virol (1988) 69:1111–6. doi: 10.1099/0022-1317-69-5-1111 2836554

[6] Stucker KM Pagan I Cifuente JO Kaelber JT Lillie TD Hafenstein S. The role of evolutionary intermediates in the host adaptation of canine parvovirus. J Virol (2012) 86:1514–21. doi: 10.1128/JVI.06222-11 PMC3264339 22114336

[7] Jager MC Tomlinson JE Lopez-Astacio RA Parrish CR Van de Walle GR. Small but mighty: old and new parvoviruses of veterinary significance. Virol J (2021) 18:210. doi: 10.1186/s12985-021-01677-y 34689822 PMC8542416

[8] François S Filloux D Roumagnac P Bigot D Gayral P Martin DP. Discovery of parvovirus-related sequences in an unexpected broad range of animals. Sci Rep (2016) 6. doi: 10.1038/srep30880 PMC5013282 27600734

[9] Calatayud O Esperón F Cleaveland S Biek R Keyyu J Eblate E. Carnivore parvovirus ecology in the serengeti ecosystem: vaccine strains circulating and new host species identified. J Virol (2019) 93. doi: 10.1128/JVI.02220-18 PMC6580958 30996096

[10] Stuetzer B Hartmann K. Feline parvovirus infection and associated diseases. Vet J (2014) 201:150–5. doi: 10.1016/j.tvjl.2014.05.027 24923754

[11] Clinical Signs I Csiza CK Scott FW Lahunta A DE Gillespie JH. Pathogenesis of feline panleukopenia virus in susceptible newborn kittens I. Clinical signs, hematology, serology, and virology. Infect Immun (1971) 3:833–7. doi: 10.1128/iai.3.6.833-837.1971 PMC416246 16558063

[12] Parrish CR Aquadro CF Carmichael LE. Canine host range and a specific epitope map along with variant sequences in the capsid protein gene of canine parvovirus and related feline, mink, and raccoon parvoviruses. Virology (1988) 166:293–307. doi: 10.1016/0042-6822(88)90500-4 3176341

[13] MIranda C Thompson G. Canine parvovirus: the worldwide occurrence of antigenic variants. J Gen Virol (2016) 97:2043–57. doi: 10.1099/jgv.0.000540 27389721

[14] Barrs VR. Feline panleukopenia: A re-emergent disease. Vet Clin North Am Small Anim Pract (2019) 49:651–70. doi: 10.1016/j.cvsm.2019.02.006 30967253

[15] Guo L Yang SL Chen SJ Zhang Z Wang C Hou R. Identification of canine parvovirus with the Q370R point mutation in the VP2 gene from a giant panda (Ailuropoda melanoleuca). Virol J (2013) 10. doi: 10.1186/1743-422X-10-163 PMC3680276 23706032

[16] Huang S Li X Xie W Guo L You D Xu H. Molecular detection of parvovirus in captive siberian tigers and lions in northeastern China from 2019 to 2021. Front Microbiol (2022) 13. doi: 10.3389/fmicb.2022.898184 PMC9133805 35633695

[17] Zhao J Zhang H Zhang L Zhang Q Zhou N Du T. Isolation and genetic characterization of parvoviruses from dogs, cats, minks, and raccoon dogs in the eastern region of shandong province, China. Front Microbiol (2022) 13. doi: 10.3389/fmicb.2022.862352 PMC8919035 35295295

[18] Guerrero-Sánchez S Wilson A González-Abarzúa M Kunde M Goossens B Sipangkui R. Serological evidence of exposure of Bornean wild carnivores to feline-related viruses at the domestic animal-wildlife interface. Transbound Emerg Dis (2022) 69:e3250–4. doi: 10.1111/tbed.14549 PMC9790233 35373926

[19] Chen CC Chang AM Wada T Chen MT Tu YS. Distribution of Carnivore protoparvovirus 1 in free-living leopard cats (Prionailurus bengalensis chinensis) and its association with domestic carnivores in Taiwan. PloS One (2019) 14. doi: 10.1371/journal.pone.0221990 PMC6719846 31479483

[20] Kim YJ Yoon SW Jang JH Jeong DG Lee BJ Kim HK. Genetic characterization of feline parvovirus isolate fe-P2 in korean cat and serological evidence on its infection in wild leopard cat and asian badger. Front Vet Sci (2021) 8. doi: 10.3389/fvets.2021.650866 PMC8138573 34026890

[21] Wessling-Resnick M. Crossing the iron gate: why and how transferrin receptors mediate viral entry. Annu Rev Nutr (2018) 38:431. doi: 10.1146/annurev-nutr-082117-051749 29852086 PMC6743070

[22] Parker JSL Murphy WJ Wang D O’Brien SJ Parrish CR. Canine and feline parvoviruses can use human or feline transferrin receptors to bind, enter, and infect cells. J Virol (2001) 75:3896–902. doi: 10.1128/JVI.75.8.3896-3902.2001 PMC114880 11264378

[23] Hueffer K Parker JSL Weichert WS Geisel RE Sgro J-Y Parrish CR. The natural host range shift and subsequent evolution of canine parvovirus resulted from virus-specific binding to the canine transferrin receptor. J Virol (2003) 77:1718–26. doi: 10.1128/JVI.77.3.1718-1726.2003 PMC140992 12525605

[24] Hueffer K Govindasamy L Agbandje-McKenna M Parrish CR. Combinations of two capsid regions controlling canine host range determine canine transferrin receptor binding by canine and feline parvoviruses. J Virol (2003) 77:10099–105. doi: 10.1128/JVI.77.18.10099-10105.2003 PMC224579 12941920

[25] Hafenstein S Palermo LM Kostyuchenko VA Xiao C Morais MC Nelson CDS. Asymmetric binding of transferrin receptor to parvovirus capsids. Proc Natl Acad Sci USA (2007) 104:6585–9. doi: 10.1073/pnas.0701574104 PMC1871829 17420467

[26] Lee H Callaway HM Cifuente JO Bator CM Parrish CR Hafenstein SL. Transferrin receptor binds virus capsid with dynamic motion. Proc Natl Acad Sci U.S.A. (2019) 116:20462–71. doi: 10.1073/pnas.1904918116 PMC6789729 31548398

[27] Callaway HM Welsch K Weichert W Allison AB Hafenstein SL Huang K. Complex and dynamic interactions between parvovirus capsids, transferrin receptors, and antibodies control cell infection and host range. J Virol (2018) 92:460–78. doi: 10.1128/JVI.00460-18 PMC6002733 29695427

[28] Cheng N Zhao Y Han Q Zhang W Xi J Yu Y. Development of a reverse genetics system for a feline panleukopenia virus. Virus Genes (2019) 55:95–103. doi: 10.1007/s11262-018-1621-9 30519855

[29] Thompson AJ de Vries RP Paulson JC. Virus recognition of glycan receptors. Curr Opin Virol (2019) 34:117–29. doi: 10.1016/j.coviro.2019.01.004 30849709 PMC6476673

[30] Neu U Bauer J Stehle T. Viruses and sialic acids: Rules of engagement. Curr Opin Struct Biol (2011) 21:610–18. doi: 10.1016/j.sbi.2011.08.009 21917445 PMC3189341

[31] Ströh LJ Stehle T. Glycan engagement by viruses: receptor switches and specificity. Annu Rev Virol (2014) 1:285–306. doi: 10.1146/annurev-virology-031413-085417 26958723

[32] Löfling J Michael Lyi S Parrish CR Varki A. Canine and feline parvoviruses preferentially recognize the non-human cell surface sialic acid N-glycolylneuraminic acid. Virology (2013) 440:89–96. doi: 10.1016/j.virol.2013.02.009 23497940 PMC3634669

